# Digital interventions to promote physical activity among inactive adults: A study protocol for a hybrid type I effectiveness-implementation randomized controlled trial

**DOI:** 10.3389/fpubh.2022.925484

**Published:** 2022-10-21

**Authors:** Paolo Zanaboni, Unn Sollid Manskow, Edvard Hamnvik Sagelv, Bente Morseth, Alf Egil Edvardsen, Inger-Lise Aamot, Bjarne Martens Nes, Bryce Hastings, Marie-Pierre Gagnon, Konstantinos Antypas

**Affiliations:** ^1^Norwegian Centre for E-health Research, University Hospital of North Norway, Tromsø, Norway; ^2^Department of Clinical Medicine, Faculty of Health Sciences, UiT The Arctic University of Norway, Tromsø, Norway; ^3^School of Sport Sciences, UiT The Arctic University of Norway, Tromsø, Norway; ^4^Memento U, Trondheim, Norway; ^5^Norwegian National Advisory Unit on Exercise Training as Medicine for Cardiopulmonary Condition, St. Olavs Hospital, Trondheim, Norway; ^6^K.G. Jebsen Center for Exercise in Medicine - CERG, Norwegian University of Science and Technology, Trondheim, Norway; ^7^Les Mills International, Auckland, New Zealand; ^8^Faculty of Nursing, Université Laval, Québec, QC, Canada; ^9^SINTEF Digital, Oslo, Norway

**Keywords:** physical activity, digital interventions, e-health, mobile health, lifestyle diseases, randomized controlled trial

## Abstract

**Introduction:**

Physical inactivity is the fourth leading risk factor for global mortality, and inactive adults have a higher risk to develop lifestyle diseases. To date, there is preliminary evidence of the efficacy of fitness technologies and other digital interventions for physical activity (PA) promotion. Intervention studies are needed to test the effectiveness and implementation of innovative PA promotion strategies.

**Methods and analysis:**

The ONWARDS study is a hybrid type I effectiveness-implementation randomized control trial aiming at an inactive and presumably high-risk population living in Northern Norway. One hundred and eighty participants will be assigned to 3 groups in a 1:1:1 ratio and participate for 18 months. Participants in group A will be provided an activity tracker with the personalized metric Personal Activity Intelligence (PAI). Participants in group B will be provided with both an activity tracker with the personalized metric PAI and access to online training videos (Les Mills+) to perform home-based training. Participants in group C will be provided an activity tracker with the personalized metric PAI, home-based online training and additional peer support *via* social media. The primary objective is to test which combination of interventions is more effective in increasing PA levels and sustaining long-term exercise adherence. Secondary objectives include: proportion of participants reaching PA recommendations; exercise adherence; physical fitness; cardiovascular risk; quality of life; perceived competence for exercise; self-efficacy; social support; usability; users' perspectives on implementation outcomes (adoption, acceptability, adherence, sustainability). The study design will allow testing the effectiveness of the interventions while gathering information on implementation in a real-world situation.

**Discussion:**

This study can contribute to reduce disparities in PA levels among inactive adults by promoting PA and long-term adherence. Increased PA might, in turn, result in better prevention of lifestyle diseases. Digital interventions delivered at home can become an alternative to training facilities, making PA accessible and feasible for inactive populations and overcoming known barriers to PA. If effective, such interventions could potentially be offered through national health portals to citizens who do not meet the minimum recommendations on PA or prescribed by general practitioners or specialists.

**Trial registration:**

https://clinicaltrials.gov/ct2/show/NCT04526444, Registered 23 April 2021, identifier: NCT04526444.

## Introduction

Physical inactivity is the fourth leading risk factor for global mortality (1). Insufficient physical activity (PA) has major implications for global health and the prevalence of lifestyle diseases, such as cardiovascular conditions, diabetes and cancer, and their associated risk factors (1, 2). Compared to inactive adults, active individuals have lower rates of all-cause mortality, lower risk to develop lifestyle diseases, lower risk of an injury, higher level of cardiorespiratory and muscular fitness, and healthier body mass and composition (3, 4).

According to the WHO 2020 guidelines on PA for health, adults aged 18–64 should perform 150–300 min of moderate-intensity PA per week, or at least 75–150 min of vigorous-intensity PA per week, or an equivalent combination, irrespective of gender, race, ethnicity or income level (5). Worldwide, nearly one third of adults are physically inactive (6). In Norway, only one third of the adult population meets the minimum recommendations on PA (7). In Northern Norway, a region characterized by a high peripherality and adverse weather conditions, PA levels are lower than in Southern Norway (8).

Inactive individuals may not know how, when, where, and with whom they can increase their PA (9). It is especially important to address the key barriers in inactive populations such as psychological factors (lack of motivation, lack of skills/knowledge), environmental obstacles (lack of facilities, bad weather), time constraints (lack of time), and social limitations (lack of social support) (9, 10). Interventions based on behavior change techniques, including goal setting, feedback and monitoring, and social support (11), have been proven to be effective at increasing PA levels in young adults in the shorter-term (12). Further research is needed to determine strategies to achieve longer-term effectiveness of PA interventions (12).

Digital technologies such as wearables, websites and mobile apps are increasingly used in interventions targeting PA together with behavior change techniques and computer-tailored methods (13, 14). Due to their broad availability, digital interventions have the potential to make PA more accessible and feasible for all groups, reduce health inequalities and support long-term adherence to PA recommendations (9, 14).

Consumer-based wearable activity trackers and smartphone apps are now widely available and may offer an alternative method for assisting individuals to remain physically active (9, 15). These devices provide individuals with the ability to objectively monitor their PA levels by features such as distance walked, number of steps, frequency or duration of activity (16) or time spent in various intensities of PA (17). Unlike other PA metrics, heart rate changes reflect the body's response to exercise regardless of the type of activity performed (16). Some apps can also provide tailored feedback through specifically designed algorithms (15). An example is the Personal Activity Intelligence (PAI), which takes into account age, sex and heart rate patterns to provide a single score indicating whether the current PA level is sufficient to obtain or sustain good health (16, 18). Despite representing a promising tool for delivering accessible and appealing PA interventions (19, 20), the evidence supporting the effectiveness of activity trackers and smartphone apps to increase PA is modest at best (15, 21–23). Moreover, there is limited insight into the sustainability of increased PA levels and long-term adherence, and further research should include long-term follow-up assessments (21).

Wearable activity trackers can be used either as the primary component of an intervention or as part of a broader PA intervention (15) which could include other useful components and behavior change techniques. Home-based exercise programs have been shown to be effective in promoting PA in low-active older adults (24). While short-terms effects seem to be achievable, poor adherence to home-based exercise can limit the long-term benefits (25). Home-based online training can provide a platform to scale effective support for sustainable behavior change, thus empowering adults to perform regular PA and increasing engagement over time (20). Participation in home exercise video programmes has been piloted and proven to be feasible (26). Peer support groups represent another well-documented technique to ensure adherence to PA among adults and maintenance of behavior change (27). Peer support provided *via* social media has the potential to support lifestyle change among young adults (28) and further increase the effectiveness of PA interventions and long-term adherence (29, 30). Moreover, the provision of educational information, simple self-monitoring strategies and regular feedback from peers challenging the individuals' capability might help people to maintain PA (31).

This hybrid type I effectiveness-implementation randomized controlled trial (RCT) aims to explore longitudinal changes in PA, long-term exercise maintenance, health and implementation outcomes of an activity tracker with the personalized metric PAI, home-based online training and peer support *via* social media among inactive adults. These digital interventions have the potential to promote PA among high-risk populations, thus resulting in better health and prevention of lifestyle diseases.

## Methods and analysis

### Study design

**Figure 1:**
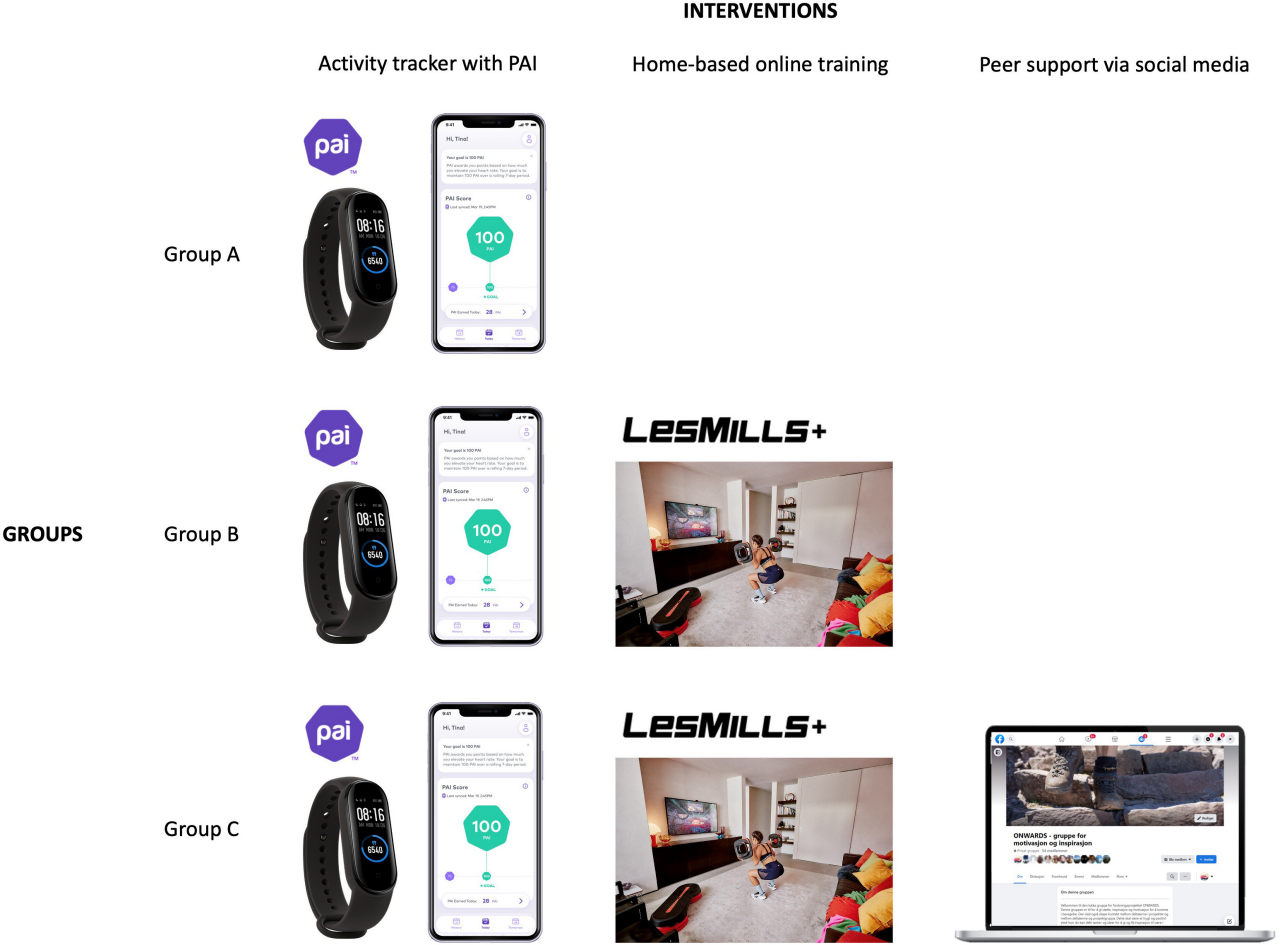
Interventions provided to the three groups.

The ONWARDS study is a hybrid Type I effectiveness-implementation RCT (32) targeting an inactive and presumably high-risk population living in the Troms and Finnmark county in Northern Norway. One hundred and eighty participants will be assigned to 3 groups in a 1:1:1 ratio and participate for 18 months. The effectiveness-implementation hybrid design allows testing the effectiveness of the interventions while gathering information on delivery and potential for implementation in a real-world situation. Participants in group A will be provided with an activity tracker with the personalized metric PAI. Participants in group B will be provided with both an activity tracker with the personalized metric PAI and access to Les Mills+ online workouts to perform home-based training. Participants in group C will be provided with an activity tracker with the personalized metric PAI, Les Mills+ online workouts and additional peer support *via* social media (Figure 1). The presence of three interventional groups will allow testing of which combination of strategies is more effective in increasing PA levels. The study is restricted to participants who volunteer and provide written informed consent in accordance with the Declaration of Helsinki. The trial received approval from the Regional Committee for Medical and Health Research Ethics (66573/REK nord). The protocol of this RCT fulfills the Standard Protocol Items: Recommendations for Interventional Trials (SPIRIT) guidelines (33) and its results will be reported according to the Consolidated Standards of Reporting Trials (CONSORT) statement (34) (Supplementary Table 1).

### Eligibility criteria

To be eligible for enrolment, participants must fulfill the following inclusion criteria: (1) young (18–40 years) or middle aged (40–55 years) healthy adults, both men and women; (2) inactive (undertaking <150 min of moderate-intensity PA per week) in the last 3 months; (3) living in the Troms and Finnmark county; (4) current owner of a smartphone; (5) able to understand training instructions in English language.

Participants will be excluded if they fulfill one or more of the following exclusion criteria: (1) presence of disabilities which might reduce the ability to perform exercise; (2) presence of conditions which might prevent from exercising safely; (3) lack of Internet connection at home; (4) lack of space to exercise safely at home (recommended 4 sqm).

### Randomization and data collection

Randomization will be web-based, stratified by sex, age, and PA level at baseline to obtain homogeneity between study groups. All data collected from the participants will be handled via REDCap (Vanderbilt University, US), a secure web platform specifically geared to support online and offline data capture for research studies. The randomization sequence will be concealed from the study team by the program. Neither study participants nor data analysts will be blinded to group allocation.

### Description of the interventions

#### Activity tracker With PAI app

Participants in groups A, B and C will be provided with an activity tracker (Mi Smart Band 5, Xiaomi, China) and use the personalized metric PAI through an app (PAI Health, Canada). PAI is a personalized metric based on robust epidemiological evidence which links personalized data to an individual's health (16, 18). PAI takes into account age, sex, resting and maximum heart rate, and analyses a continuous stream of heart rate data acquired from the user to provide a single score indicating whether the current PA level is sufficient to obtain or sustain good health (16). A score of ≥100 weekly PAI has been shown to reduce the risk of premature death due to cardiovascular disease in healthy adults as well as individuals with known risk factors, regardless of whether or not the current PA recommendations were met (18). Moreover, a PAI score ≥100 at baseline, maintaining ≥100 PAIs and an increasing PAI score over time was associated with lower mortality risk (35). Heart rate and accelerometery data are recorded and stored automatically on a daily basis, and an individual feedback on the current PAI score is provided through the smartphone app. This also works as a reminder to keep a weekly PAI score above 100 for disease prevention and health promotion.

All participants will also receive information on recommendations for PA, together with a 6-week acclimatization programme consisting of aerobic activity (two times per week) and muscle-strengthening (one time per week). For aerobic activity, information will be provided on how to exercise in moderate intensity (participants begin to sweat but can speak) including warm up, duration and intensity, examples of activities (e.g., jogging, running, cycling, cross-country skiing, swimming, ball sports, martial arts) and how to make progress in the programme. Participants will be also introduced to high-intensity (hard effort, participants cannot speak) interval training with an example of a 4 × 4 program consisting of four bouts of 4-min high-intensity training and 3-min cool-down. For muscle-strengthening, participants will be provided with an example of programme consisting of five exercises (for legs, back, arms and shoulders) be performed with four sets of four repetitions, and instructed on how to perform each exercise (description and link to a video) and make progress by increasing load.

#### Online training

Participants in groups B and C will have personal access to Les Mills+ (formerly named Les Mills On Demand), an online solution offering videos of training classes available 24/7 *via* a website (https://www.lesmills.com). Workouts include cardio, strength, flexibility, core, and high-intensity training. New releases are available every 3 months to increase motivation, old releases are available to increase variety. Training with other family members or friends at home will be allowed. Les Mills+ is accessible from any device, smart TV with Internet access, pc, laptop, tablet or smartphone.

#### Peer support via social media

Participants in group C will be invited to join a Facebook closed-group. The aim of this group is to provide a platform for participants to share their experiences, ask for advice, support or motivation from peers, and discuss technical or practical challenges. The project team, as administrators, will provide general information and educational advice about PA, motivational support, rewarding messages, technical and practical help. This, in turn, might prevent dropouts.

### Recruitment and study procedures

**Figure 2:**
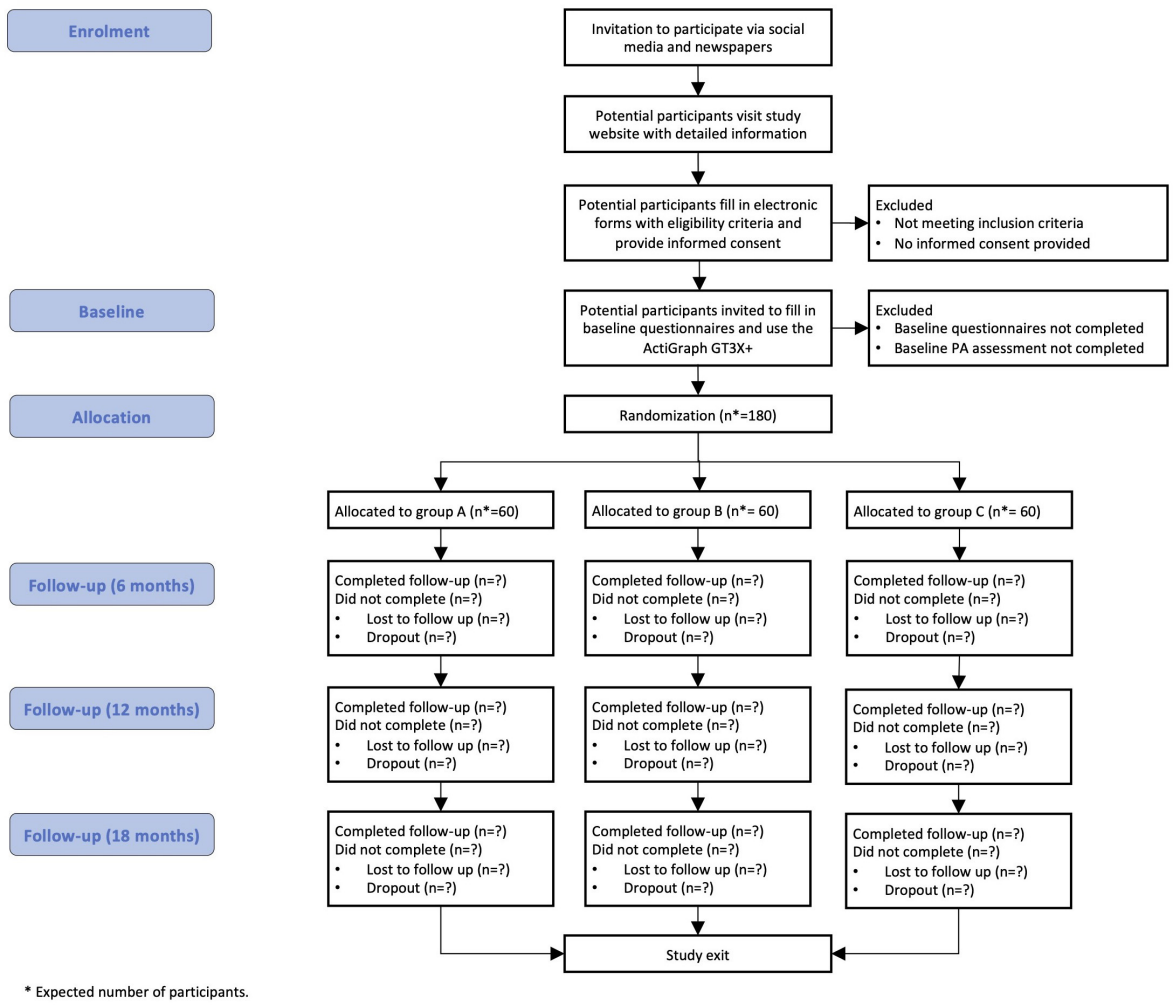
Flow of study participants.

Potential participants will be invited primarily *via* Facebook advertised campaigns designed to address adults who fit the inclusion criteria (Figure 2). Advertisement through local newspapers will also be used. People will express their interest, complete an online form including questions on the eligibility criteria (answers will be self-reported), and sign an electronic informed consent. Eligible participants will receive information about the study by e-mail and have a discussion with the research team if needed.

At enrolment, participants will receive the ActiGraph GT3X+ accelerometer by mail, asked to wear it for eight consecutive days and return it by a prepaid mail envelope. Participants will also be asked to measure their waist circumference and complete the online study questionnaires. Participants will then be randomized to one of the three groups and be informed about which group they have been assigned to.

After enrolment, all participants will receive the activity tracker Mi Smart Band 5 directly to their homes and install the PAI Health app on their smartphone. Detailed instructions on how to use the activity tracker and the PAI Health app will be sent to each participant by email, together with together with a 6-week acclimatization programme. Participants in groups B and C will receive personal credentials to access to Les Mills+. Participants in group C will be invited to a Facebook group used for peer support.

At 6-, 12-, and 18-month participants will undergo a follow-up, asked to wear the ActiGraph GT3X+ accelerometer for eight consecutive days, measure their waist circumference, and complete the online study questionnaires. Qualitative interviews will be conducted in a subsample of participants (*n* = 18–20) at 6- and 18-month to explore their perception of the implementation of the interventions. During data collection, participants will be encouraged to continue participating in the study. A gift card (NOK 300) will be sent to each participant after completion of the 6-month follow up to prevent attrition. Data collection for all follow-ups will be performed remotely, as no clinical examinations are planned at any time point. Study end is set 18 months after enrolment.

### Study objectives and hypotheses

The primary objective of this study is to test which combination of digital interventions is more effective in increasing PA levels among physically inactive adults and sustaining long-term exercise adherence. The secondary objective is to determine the effects on health and implementation outcomes, including: proportion of participants reaching PA recommendations; exercise adherence; physical fitness; cardiovascular risk; quality of life; perceived competence for exercise; self-efficacy; social support; usability; users' perspectives on implementation outcomes (adoption, acceptability, adherence, sustainability).

We hypothesize that the use of activity trackers with the personalized metric PAI can promote PA and improve health outcomes. Furthermore, we hypothesize that the additional use of home-based online training is more effective in engaging in regular PA. Finally, we hypothesize that participation in peer support group *via* social media will result in better long-term adherence to PA.

### Outcome measures

The primary outcome is the average time spent in moderate and vigorous PA. Objective measurement of PA will be undertaken using the ActiGraph GT3X+ accelerometer worn at the right hip by each participant for eight consecutive days. The ActiGraph GT3X+ should be taken off when showering or bathing. A day of data will be included if there are at least 10 h of data. A minimum of four valid days of data will be required (36). Total physical activity will be measured as total acceleration counts per minute in triaxal vector magnitude (square root of the summed activity counts from all three axes) counts per minute. The intensity of PA will be split by acceleration cut-off of <150 triaxial counts per minute (sedentary time), 150–2,689 triaxial counts per minute for light and >2,690 triaxial counts per minute for moderate and vigorous PA (37, 38), which are calibrated to correspond to <1.5, 1.5–2.9 and 3.0 metabolic equivalents of tasks (METs).

The proportion of participants reaching the recommendation for PA will be measured, for each group, at all time points (Table 1). Longitudinal changes will express a measure of maintenance of long-term adherence to exercise. The proportion of participants reaching the goal of 100 PAI/week will also be measured for the entire duration of the study.

**Table 1:** Schedule of enrolment, interventions, and assessments.

**Time point**	**Enrolment**	**Baseline**	**6-month**	**12-month**	**18-month**
**Enrolment**
Eligibility criteria	X				
Informed consent	X				
Allocation		X			
**Interventions**
Group A					
Group B					
Group C					
**Assessments**
Demographic data		X			
Physical activity level		X	X	X	X
Adherence to PA recommendation		X	X	X	X
Physical fitness (VO_2max_)		X	X	X	X
BMI		X	X	X	X
Waist circumference		X	X	X	X
Quality of life (SF-12)		X	X	X	X
Perceived competence for exercise (PCS)		X	X	X	X
Self-efficacy for exercise (SEE)		X	X	X	X
Social Support (SSES)		X	X	X	X
Reasons for performing physical activity		X	X	X	X
Usability (SUS)			X		
Users' perspectives (interviews)			X		X

*PA, physical activity; VO*_2*max*_*, maximal oxygen uptake; BMI, body mass index; SF-12, 12-Item Short Form Survey; PCS, perceived competence scale; SEE, Self-Efficacy for Exercise Scale; SSES, Social Support and Exercise Survey; SUS, System Usability Scale*.

The maximal oxygen uptake (VO_2max_) is the most precise measure of cardiovascular fitness and represents the body's capacity to transport and use oxygen during exercise. In this study, VO_2max_ will be estimated from a validated non-exercise prediction model which includes age, waist circumference, resting heart rate and leisure-time PA (39, 40). The algorithm used to estimate cardiorespiratory fitness is also integrated and freely available in a publicly accessible online tool (www.worldfitnesslevel.org). One of the co-authors (BMN) holds the Intellectual Property rights for this tool, which is available for commercial actors upon license agreements.

Cardiovascular risk will be assessed by the following risk factors: smoking, body mass index (BMI), and waist circumference. Height, weight and waist circumference will be self-measured by participants and self-reported on the online study questionnaires.

Quality of life will be measured with the 12-Item Short Form Survey (SF-12) (41). Perceived competence will be measured with the perceived competence scale (PCS) for regular physical exercise (42). Self-efficacy will be measured with the Self-Efficacy for Exercise Scale (SEE) (43). Social support for PA from friends and family will measured using the Social Support and Exercise Survey (SSES) (44). Usability of the interventions will be assessed at 6-month only with the System Usability Scale (SUS) (45). Reasons for performing and not performing physical activity will be collected with multiple-choice questionnaires previously used in Norwegian surveys (7).

Users' perspectives will be explored with semi-structured interviews conducted in a subsample of participants at 6- and 18-month to explore their perception on implementation outcomes including adoption, acceptability, adherence and sustainability of the interventions (46). Interviews will be recorded on audio digital file, transcribed verbatim and analyzed *via* Nvivo upon the theoretical frame of the learning theory.

### Statistical analysis

Descriptive statistics will be reported as mean and standard deviation for normally distributed continuous variables, or median with interquartile range in the case of skewed distribution. An intention-to-treat analysis will be performed on all randomized subjects to provide unbiased comparisons among groups and avoid the effects of dropout. Study outcomes will be measured as changes from baseline to all assessment points (6-, 12-, 18-month). Changes will be tested with linear mixed models, which account for repeated measures collected in a longitudinal design and deal better with dropouts, without the need for imputation of missing data. Intraclass correlations coefficients will be also reported to take into account the potential correlation among individuals within group. Statistical analyses will be performed with IBM SPSS Statistics.

### Sample size

The sample size is based on the number of participants needed to detect significant longitudinal changes in PA level. With a statistical power of 0.80, an alpha value of 0.05, a moderate correlation (*r* = 0.5) between measures, and an expected 10% attrition, a total sample size of 180 participants (60 per group) is required to detect a medium effect size (*d* = 0.53) (21). Calculation was performed by a statistician.

## Discussion

The ONWARDS study aims to explore longitudinal changes in PA, long-term exercise maintenance, health and implementation outcomes among inactive adults using three different digital interventions for PA promotion, namely an activity tracker with the personalized metric PAI, home-based online training *via* Les Mills+ and peer support *via* a Facebook closed-group. Inactive adults often do not access a training facility or use equipment for home exercise. A control group receiving “standard care” was therefore deemed to be unfair for these individuals. All participants in this study will therefore be provided with at least an activity tracker. The presence of three interventional groups will allow testing of which combination of strategies is more effective in increasing PA levels and maintaining them over the long-term.

Psychological factors, including lack of motivation and lack of skills/knowledge, represent a major barrier to PA (9, 10). Perceived self-efficacy can affect both motivation and actions and is important for lasting changes in PA (47). Other effective behavior change techniques used in mobile apps for PA include goal setting, monitoring and feedback about whether or not one gets enough exercise (13). PAI is personalized metric based on heart rate data which can be used by everyone of all ages and fitness levels. The PAI Health mobile app sets a clear goal, which is to keep a weekly PAI score above 100 for disease prevention and health promotion. Users who struggle with keeping physically active can self-monitor their PA level *via* the PAI Health app, which provides a daily feedback on their current PAI score and reminds them to try to reach their goal. Thanks to these features, we expect that the participants in this study will increase their perceived self-efficacy, competence and motivation for exercise which, in turn, might result in higher PA levels and better adherence to PA recommendation.

Geographical isolation and related environmental factors (e.g., lack of facilities) are especially related to health disparities and inequality in PA (48). Access to training facilities can promote PA with equipment for strength and aerobic exercise as well as group-based classes, which are motivating and result in better long-term adherence (29). However, many people do not have access to these facilities, and others have concerns about their appearance (10). Adverse weather conditions represent another major barrier to participation in PA, and high or low temperatures, rain, snow or wind may all decrease the pleasure derived from outdoor activities (49). Lack of time is one of the most reported reasons why healthy adults do not participate in sport or physical recreation (10, 50). Northern Norway, where this study will be conducted, is a region characterized by frequent adverse weather conditions as well as a high peripherality, with several people living outside urban areas and without easy access to training facilities. Moreover, study participants will be inactive young or middle aged adults, which most likely will be studying or working and might therefore perceive lack of time as a barrier to PA. The possibility for participants in groups B and C to exercise conveniently from home supported by video programmes has the potential to empowering them to perform regular PA while addressing common issues such as lack of time or concerns about appearance. Moreover, interventions with a group atmosphere, such as Les Mills+ online programmes, can be more effective in increasing engagement over time and reducing perceived stress (20).

The lack of social support is another common barrier to PA (10). Poor adherence to home-based exercise (24), in addition to the poor adherence observed in e-health interventions (51), makes the implementation of digital technologies for PA a challenging issue. Novel strategies are needed to ensure adherence to PA among adults and maintenance of behavior change (27). Social media are nowadays widely used in many people's daily routines and are shown to be valuable for targeting lifestyle change among young adults (28). In a study examining the efficacy of a Facebook social support group to increase PA in young women, participants in a Facebook social support group increased the number of steps per day more than those in the standard walking intervention (30). Participants in group C will be offered peer support *via* a Facebook closed-group, where they will have the possibility to share their experiences or ask for advice from other members of the group, as well as receive educational information about PA, motivational support and rewarding messages from the project team. It is expected that peer-support *via* social media will result in better adherence to PA, better maintenance of PA levels over the long-term, as well as fewer dropouts.

This study can contribute to reduce disparities in PA levels among inactive adults by increasing access to PA and promoting long-term adherence. Increased PA might, in turn, result in better prevention of lifestyle diseases. Digital interventions delivered at home can become an alternative to training facilities, making PA accessible and feasible for inactive populations and overcoming known barriers to PA. If effective, such interventions could potentially be offered on a large-scale through national health portals to all citizens who do not meet the minimum recommendations on PA. Effective e-health interventions for PA could also be prescribed by general practitioners or specialists to both healthy and impaired individuals. The study will be conducted in the Troms and Finnmark county in Northern Norway. However, the results will be applicable to other regions and countries. The interventions could be also applicable to selected patient groups, particularly those with mobility impairment characterized by low levels of PA.

## Ethics statement

The studies involving human participants were reviewed and approved by Regional Committee for Medical and Health Research Ethics (66573/REK nord). The patients/participants provided their written informed consent to participate in this study.

## Author contributions

Procured funding: PZ. Conceptualization, design, and critical review of manuscript: PZ, USM, EHS, BM, AEE, I-LA, BMN, BH, M-PG, and KA. Drafting manuscript: PZ, USM, EHS, and KA. All authors have read and approved the manuscript.

## Funding

This study was funded by the Northern Norway Regional Health Authority (Project Grant HNF1428-18). The study protocol has undergone peer-review by the funding body. Access to Les Mills+ for the participants in groups B and C was provided for free by Les Mills International (Auckland, New Zealand).

## Conflict of interest

Author AEE was employed by Memento U. Author BH is Head of Research at Les Mills International. The remaining authors declare that the research was conducted in the absence of any commercial or financial relationships that could be construed as a potential conflict of interest.

## Publisher's note

All claims expressed in this article are solely those of the authors and do not necessarily represent those of their affiliated organizations, or those of the publisher, the editors and the reviewers. Any product that may be evaluated in this article, or claim that may be made by its manufacturer, is not guaranteed or endorsed by the publisher.

## Abbreviations

PA: physical activity
PAI: Personal Activity Intelligence
RCT: randomized controlled trial
SPIRIT, Standard Protocol Items: Recommendations for Interventional Trials
MET: metabolic equivalent
VO_2max_: maximal oxygen uptake
BMI: body mass index
SF-12: 12-Item Short Form Survey
PCS: perceived competence scale
SEE: Self-Efficacy for Exercise Scale
SSES: Social Support and Exercise Survey
SUS: System Usability Scale.
